# Combination therapy with androgen receptor N‐terminal domain antagonist EPI‐7170 and enzalutamide yields synergistic activity in AR‐V7‐positive prostate cancer

**DOI:** 10.1002/1878-0261.12770

**Published:** 2020-08-09

**Authors:** Yukiyoshi Hirayama, Teresa Tam, Kunzhong Jian, Raymond J. Andersen, Marianne D. Sadar

**Affiliations:** ^1^ Genome Sciences Centre BC Cancer Vancouver BC Canada; ^2^ Department of Chemistry University of British Columbia Vancouver BC Canada; ^3^ Department of Earth, Ocean, and Atmospheric Sciences University of British Columbia Vancouver BC Canada; ^4^ Department of Pathology and Laboratory Medicine University of British Columbia Vancouver BC Canada

**Keywords:** androgen receptor, AR‐V7, combination therapy, CRPC, EPI‐002, ralaniten

## Abstract

Resistance of castration‐resistant prostate cancer (CRPC) to enzalutamide and abiraterone involves the expression of constitutively active, truncated androgen receptor (AR) splice variants (AR‐Vs) that lack a C‐terminal ligand‐binding domain (LBD). Both full‐length AR and truncated AR‐Vs require a functional N‐terminal domain (NTD) for transcriptional activity thereby providing rationale for the development of ralaniten (EPI‐002) as a first‐in‐class antagonist of the AR‐NTD. Here, we evaluated the antitumor effect of a next‐generation analog of ralaniten (EPI‐7170) as a monotherapy or in combination with enzalutamide in prostate cancer cells that express AR‐V7 that were resistant to enzalutamide. EPI‐7170 had 8–9 times improved potency compared to ralaniten. Enzalutamide increased levels of AR‐V7 and expression of its target genes. Knockdown of AR‐V7 restored sensitivity to enzalutamide, indicating a role for AR‐V7 in the mechanism of resistance. EPI‐7170 inhibited expression of genes transcriptionally regulated by full‐length AR and AR‐V7. A combination of EPI‐7170 and enzalutamide resulted in synergistic inhibition of proliferation of enzalutamide‐resistant cells that was consistent with results from cell cycle and clonogenic assays. In addition, this drug enhanced the antitumor effect of enzalutamide in enzalutamide‐resistant CRPC preclinical models. Thus, a combination therapy targeting both the NTD and LBD of AR, and thereby blocking both full‐length AR and AR‐Vs, has potential for the treatment of enzalutamide‐resistant CRPC.

## Graphical abstract

Ralaniten and analogs (EPI) bind androgen receptor N‐terminal domain (AR‐NTD) to block the transcriptional activities of full‐length AR (FL‐AR) and AR splice variants (AR‐Vs). Enzalutamide (ENZ) elevates levels of AR‐V7 leading to resistance and increased proliferation. Targeting AR‐NTD to block FL‐AR and AR‐Vs with EPI in combination with ENZ resulted in synergistic inhibition of proliferation of ENZ‐resistant prostate cancer cells.

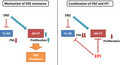

**Abbreviations**
AR: androgen receptor
AR‐V7: androgen receptor splice variant‐7
CRPC: castration‐resistant prostate cancer
ENZ: enzalutamide
FL‐AR: full‐length androgen receptor
LBD: ligand‐binding domain
NTD: N‐terminal domain

## 1 Introduction

Abiraterone and second‐generation antiandrogens such as enzalutamide (ENZ) target full‐length (FL) androgen receptor (AR) C‐terminal ligand‐binding domain (LBD). These AR‐LBD inhibitors have demonstrated significant responses for castration‐resistant prostate cancer (CRPC). Unfortunately the majority of these patients eventually develop adaptive resistance following an initial response [1, 2, 3, 4]. In efforts to improve outcomes, the PLATO trial tested the addition of abiraterone to ongoing ENZ and revealed that this combination does not improve progression‐free survival for CRPC patients, thereby showcasing the limitations of combining AR‐LBD‐targeted therapies [5].

A key mechanism underlying lethal CRPC is the expression of constitutively active splice variants of androgen receptor (AR‐Vs) [6, 7, 8, 9]. AR‐Vs lack LBD, but retain its transactivating N‐terminal domain (NTD) that is essential for AR transcriptional activity [10, 11]. Of the many AR‐Vs that have been discovered, the most clinically relevant is AR‐V7 [6]. AR‐V7 is proposed as a predictive biomarker to direct treatment selections for CRPC patients due to its association with resistance to AR‐LBD‐targeted therapies and poor outcomes [12, 13, 14, 15]. Unfortunately, treatment strategies for AR‐V7‐positive patients are currently limited. One approach that has promise is the development of inhibitors to AR‐NTD. Recently, the first‐in‐class AR‐NTD inhibitor, ralaniten (EPI‐002), showed signs of efficacy in a phase 1 clinical trial in heavily pretreated CRPC patients that had failed abiraterone and/or ENZ [16, 17]. Due to the poor pharmacokinetics of ralaniten, the trial was stopped and the drug was redesigned for better metabolic stability and improved potency [16, 17]. Second‐generation analogs have now been developed with clinical trials expected to begin in the first half of 2020 (NCT04421222).

Ralaniten and its analogs specifically inhibit the AR‐NTD to block the transcriptional activities of full‐length AR (AR‐FL) and AR‐Vs [18, 19, 20]. Ralaniten directly binds to transactivation unit‐5 (tau‐5) in the NTD [21] to block necessary protein–protein interactions with the basal transcriptional machinery [18]. Ralaniten is the first drug that directly binds to any intrinsically disordered region/protein to be tested in clinical trials, thereby emphasizing the hurdles of developing drugs to these difficult targets [17]. In this study, we examined how AR‐V7 drives resistance to ENZ and evaluated the therapeutic efficacy of a combination of a next‐generation AR‐NTD antagonist, EPI‐7170 with ENZ as a near‐term potential therapeutic option for the treatment of CRPC.

## 2 Methods

### 2.1 Cell lines and reagents

VCaP cells were purchased from the American Type Culture Collection (Manassas, VA, USA). LNCaP cells were from L. Chung (Cedars‐Sinai Medical Center, Los Angeles, CA, USA). C4‐2B and C4‐2B enzalutamide‐resistant (C4‐2B‐ENZR) cells were from A. Gao (University of California, Davis, CA, USA) [22]. VCaP‐ENZR cells were developed by long‐term cultivation with increasing concentrations of ENZ (0.1–10 µm) beginning in November, 2017 (media supplemented with ENZ was replenished every 3–4 days). VCaP‐C cells were developed by exposure to the corresponding concentration of DMSO as an appropriate control. VCaP cells were cultured in phenol red‐free DMEM supplemented with 10% FBS, LNCaP, and C4‐2B cells in phenol red‐free RPMI‐1640 supplemented with 10% FBS, VCaP‐ENZR cells in phenol red‐free DMEM supplemented with 10% FBS and 10 µm ENZ, C4‐2B‐ENZR cells in phenol red‐free RPMI‐1640 supplemented with 10% FBS and 20 µm ENZ. All cells were grown in a humidified incubator with 5% CO_2_ at 37 °C and maintained in culture for not more than 10 passages after resurrection. All cells were regularly tested to ensure they were mycoplasma‐free and were authenticated by short tandem repeat analysis (Centre for Applied Genetics, Hospital for Sick Children, Toronto, ON, Canada). Cells were treated in phenol red‐free and charcoal stripped FBS (CSS) condition followed by androgen stimulation in assays to measure androgen‐induced signal.

Ralaniten was provided by NAEJA (Edmonton, AB, Canada), and EPI‐7170 was synthesized by us. Enzalutamide was purchased from OmegaChem (Lévis, QC, Canada) and synthetic androgen (R1881) from AK Scientific Inc (Mountain View, CA, USA).

### 2.2 Western blot analysis

Cells (VCaP, VCaP‐C, VCaP‐ENZR, C4‐2B, and C4‐2B‐ENZR) were plated in 6‐well plates in ENZ‐free media for 2 days prior to harvesting for protein isolation. For experiments for cell cycle protein, C4‐2B‐ENZR cells were plated in 6‐cm dishes and treated with vehicle, ENZ (20 µm), EPI‐7170 (2.5 or 3.5 µm), or its combination in phenol red‐free RPMI‐1640 plus 1.5% FBS for 48 h and harvested for protein isolation. Protein was extracted with RIPA buffer and protease inhibitor cocktails (Roche Diagnostics, Laval, QC, Canada). The amount of protein was determined using a BCA protein assay kit (Pierce, Rockford, IL, USA). Equal amounts of denatured proteins were separated by SDS/PAGE gels and transferred to a PVDF membrane and blocked with 5% skim milk in PBS containing 0.1% Tween 20. The membranes were probed with the following primary antibodies: AR (N‐20) (ab108341) (Abcam, Cambridge, MA, USA), AR‐V7 (RM7) (RevMAb Biosciences, South San Francisco, CA, USA), Cyclin D1 (#2978) diluted 1 : 1000, Cyclin A2 (#4656) diluted 1 : 1000, CDK4 (#12790) (Cell Signaling Technology, Danvers, MA, USA) diluted 1 : 1000, and Actin (A5441) (Sigma‐Aldrich, Oakville, ON, Canada) diluted 1 : 10 000.

### 2.3 siRNA transfection and proliferation assays

siRNA against AR exon3b was obtained from Dharmacon (Lafayette, CO, USA). The target sequence for AR exon3b is 5′‐GUAGUUGUGAGUAUCAUGA‐3′. siRNA knockdown was performed using Lipofectamine RNAiMAX Transfection Reagent (Invitrogen, Carlsbad, CA, USA) according to the manufacturer's protocol. VCaP‐ENZR (5 × 10^5^ cells) and C4‐2B‐ENZR (3 × 10^5^ cells) cells were, respectively, plated and incubated for 24 h. siRNA/transfection reagent complexes were added into each wells. Forty‐eight hours post‐transfection, protein was harvested and analyzed by western blot analysis.

For experiments combining drug treatment with siRNA transfection, VCaP‐ENZR (8 × 10^4^ cells/well) and C4‐2B‐ENZR (2 × 10^4^ cells/well) were plated in 96‐well plates and transfected with siRNA 24 h prior to treatment with enzalutamide for 5 days (VCaP‐ENZR) or 3 days (C4‐2B‐ENZR). BrdU incorporation was measured using BrdU ELISA kit (Roche Diagnostics, Laval, QC, Canada).

### 2.4 Quantitative real‐time PCR

VCaP‐ENZR and C4‐2B‐ENZR cells were pretreated for 1 h with vehicle, EPI‐7170, ENZ, or a combination before addition of 0.1 nm R1881 under 1.5% CSS and phenol red‐free conditions and then incubated for another 48 h. RNA was extracted using TRIzol (Invitrogen) and PureLink RNA Mini Kit (Life Technologies, Waltham, MA, USA) according to the manufacturer's protocol. Reverse transcription reactions were conducted using cDNA with High Capacity RNA‐to‐cDNA kit (Applied Biosystems, Beverly, MA, USA). Quantitative real‐time RT–PCR was performed using Platinum SYBR Green qPCR SuperMix‐UDG with ROX (Invitrogen) and QuantStudio 6 Flex (Applied Biosystems). Data were normalized to RPL13a gene expression. Primers have been described previously [18, 23].

### 2.5 Sequencing FL‐AR

Total RNA (1 μg) from VCaP‐ENZR cells was reverse transcribed using SuperScript IV VILO Master Mix (Invitrogen). The coding region of AR was PCR amplified with Platinum DNA Taq Polymerase High Fidelity in 20 µL with 1 U Taq polymerase, 2 mm MgSO, 0.2 mm deoxynucleotide mix, and 2% DMSO with the AR‐NTD and LBD primers (Set#1 NTD forward:: 5′‐AGGGGAGGCGGGGTAAGGGAAGTA‐3′; reverse: 5′‐CTGGGTTGTCTCCTCAGTGGGGC‐3′ Set#2 LBD forward: 5′‐GCGAAATGGGCCCCTGGATGGAT‐3′; reverse 5′‐CATGAGCTGGGGTGGGGAAATAGG‐3′). The PCR fragment was gel purified and cloned into PCR 2.1 TOPO cloning vector using TOPO TA cloning kit from Invitrogen and transformed into chemically competent TOP10 cells. Plasmids were sequenced at the Sequencing and Bioinformatics Consortium at The University of British Columbia (Vancouver, BC, Canada; http://dnalims.sequencing.ubc.ca).

### 2.6 Proliferation assay

VCaP cells (8 × 10^3^ cells/well), VCaP‐C cells (8 × 10^3^ cells/well), VCaP‐ENZR cells (1.2 × 10^4^ cells/well), C4‐2B cells (3 × 10^3^ cells/well), and C4‐2B‐ENZR (3 × 10^3^ cells/well) cells were plated in 96‐well plates in their respective media (DMEM plus 3% FBS for VCaP, VCaP‐C and VCaP‐ENZR, RPMI‐1640 plus 1.5% FBS for C4‐2B and C4‐2B‐ENZR). After incubating for 24 h (C4‐2B and C4‐2B‐ENZR) or 48 h (VCaP and VCaP‐ENZR), cells were treated with vehicle, EPI‐7170, ENZ, or a combination for 2 days (C4‐2B and C4‐2B‐ENZR) or 5 days (VCaP and VCaP‐ENZR). Cell proliferation assays were performed using the BrdU ELISA kit (Roche Diagnostics) according to the manufacturer's protocol. Absorbance was measured with a VersaMax ELISA microplate reader (Molecular Devices, San Jose, CA, USA). The combination index (CI) values calculated by Chou‐Talalay method using compusyn software (ComboSyn, Inc., Paramus, NJ, USA ) where a CI value below 1 represents synergism.

### 2.7 Reporter gene assay

LNCaP cells were plated in 24‐well plates and incubated for 24 h. Cells were cotransfected with PSA(6.1 kb)‐luciferase reporter or V7BS_3_‐luciferase reporter and AR‐V7 expression vector or pGL4.28 empty vector under serum‐free and phenol red‐free conditions. After 24 h, cells were pretreated for 1 h with vehicle, ENZ, EPI‐7170, or a combination before addition of 1 nm R1881 and then incubated for another 24 h. Cells were harvested, and relative luminescence units (RLU) in cell lysates were detected using Promega GloMax‐Multi Detection Luminometer (Promega, Madison, WI, USA). Protein concentrations of the cell lysates were determined by the Bradford method using Bio‐Rad Protein Assay Kit (Mississauga, ON, Canada). Luciferase activities were normalized to protein concentration.

### 2.8 Flow cytometry

C4‐2B‐ENZR cells (3.5 × 10^5^ cells/plate) were plated in 6‐cm dishes and treated with vehicle, ENZ (20 µm), EPI‐7170 (2.5 or 3.5 µm), or its combination in RPMI‐1640 plus 1.5% FBS for 48 h. Cells were labeled with 10 μm BrdU for 2 h and fixed in 70% ethanol. BrdU‐labeled cells were probed with anti‐BrdU‐FITC antibody (BD Biosciences, Franklin Lakes, NJ, USA), and DNA was stained with 7‐aminoactinomycin D (Sigma‐Aldrich). Data were acquired using a FACS Calibur (BD Biosciences), and data were analyzed using flowjo software (Asland, OR, USA).

### 2.9 Colony formation assay

C4‐2B‐ENZR cells (400 cells/well) were plated in 6‐well culture plates, and after 24 h, cells were treated with ENZ (20 µm), ralaniten (25 µm), EPI‐7170 (2.5 µm), or a combination and then monitored for a colony formation for 2 weeks. Colonies were fixed with 4% paraformaldehyde and stained with 0.01% crystal violet. Colonies containing more than 50 cells were counted using imagej software version 1.45s (Rasband, W.S., ImageJ, U.S. National Institutes of Health, Bethesda, MD, USA).

### 2.10 Animal studies

The animal experiment protocols were approved by the University of British Columbia Animal Care Committee. Six‐ to eight‐week‐old male NOD/SCID mice were maintained in the Animal Care Facility at the British Columbia Cancer Research Centre. VCaP‐ENZR cells (5 × 10^6^ cells per injection) were suspended in 100 µL of PBS: Matrigel (Corning, NY, USA) (1 : 1) and subcutaneously injected into the flanks of mice. Mice were castrated when the tumors reached approximately 80 mm^3^ in volume. EPI‐7170 and ENZ were prepared in 5% DMSO, 10% Tween 80, and 1% carboxymethylcellulose. After 7 days, mice were divided into four groups and treated: control group (vehicle), ENZ group (20 mg·kg^−1^ body weight), EPI‐7170 group (30 mg·kg^−1^ body weight), and combination treatment group (concurrent treatment with ENZ and EPI‐7170). Body weight was measured every day, and tumor volume was measured twice a week. The tumor volume was calculated using the formula length × width × height × 0.52. After 31 days of treatment, the mice were euthanized and the tumors were processed for western blot analyses and IHC.

### 2.11 Immunohistochemical studies

Immunohistochemistry was performed in 5‐µm‐thick formalin‐fixed paraffin‐embedded (FFPE) sections. Sections were deparaffinized and rehydrated. Endogenous peroxidase was blocked with 3% hydrogen peroxide in distilled water for 5 min. Incubation with anti‐Ki‐67 (Dako/Agilent Technologies, Mississauga, ON, Canada) diluted in 1 : 50 was done at 4 °C overnight. Antigen was detected with 3,3‐diaminobenzidine and counterstaining with hematoxylin. For TUNEL staining, the ApopTag Fluorescein In Situ Apoptosis Detection Kit (Millipore, Billerica, MA, USA) was used according to the manufacturer's instructions.

### 2.12 Statistical analysis

Differences in the quantitative data, which are expressed as mean ± SEM, between groups were compared by one‐way ANOVA Tukey's multiple comparisons test using graphpad prism 7 (GraphPad Software, Inc., La Jolla, CA, USA). *P*‐values < 0.05 were considered statistically significant.

## 3 Results

### 3.1 Enzalutamide‐resistant prostate cancer cells express higher levels of AR‐V7

**Fig. 1:**
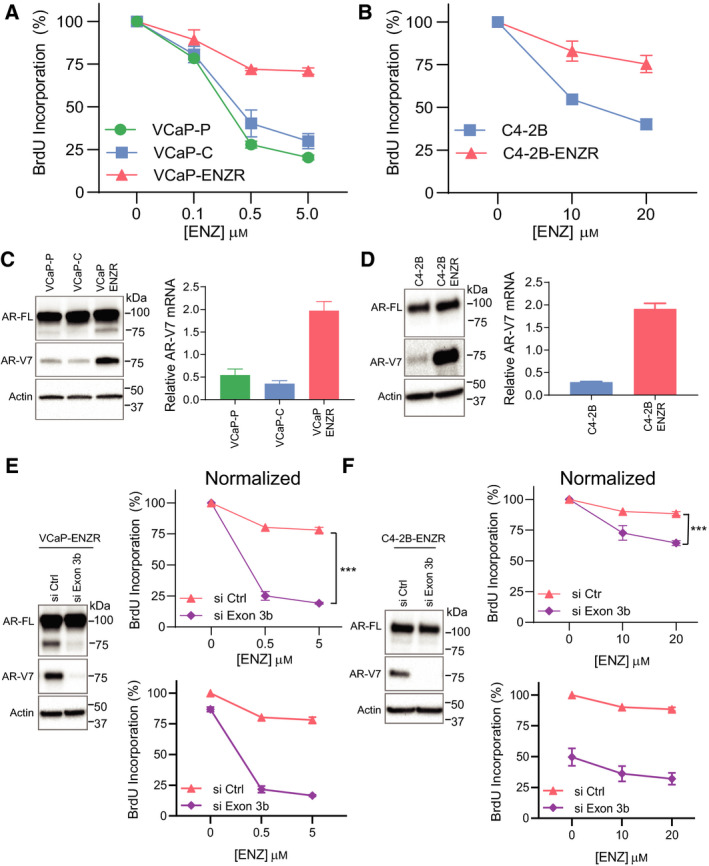
AR‐V7 confers resistance to ENZ. (A) VCaP cells were treated with different concentrations of ENZ for 5 days, and (B) C4‐2B cell lines for 2 days. Cell proliferation was analyzed by BrdU incorporation assay. Values represent the mean ± SEM (*n* = 3). (C) Levels of AR and AR‐V7 proteins and mRNAs were detected by western blot analysis and real‐time qPCR in VCaP and (D) C4‐2B cells. Relative AR‐V7 mRNA values represent the mean ± SEM (*n* = 3). (E) VCaP‐ENZR and (F) C4‐2B‐ENZR cells were transfected with control siRNA or siRNA targeting exon 3b. Levels of AR and AR‐V7 proteins were analyzed by western blot analysis. After 24 h of transfection, cells were treated with ENZ for 5 days (for VCaP‐ENZR) or 3 days (for C4‐2B‐ENZR) and cell proliferation was analyzed by BrdU incorporation assay. Data are presented normalized (top) and not normalized (bottom). Unpaired two‐tailed Student's *t*‐test. ***P < 0.001. Values represent the mean ± SEM (*n* = 3). VCaP‐P: parental VCaP; VCaP‐C: a vehicle control developed by culturing VCaP‐P with the corresponding concentration of DMSO; VCaP‐ENZR: VCaP with acquired resistance to ENZ. A representative western blot is shown from *n* = 3 independent experiments for ‘D’, ‘E’ and ‘F’ with the one exception in ‘C’ for VCaP cell lines where *n* = 2.

The role of AR‐V7 in the mechanism of resistance to ENZ was first tested in two different ENZ‐resistant prostate cancer cell lines, VCaP‐ENZR and C4‐2B ENZR. VCaP‐ENZR was established by exposing parental VCaP (VCaP‐P) cells to increasing doses of ENZ (up to 10 µm) over a period of 10 months. ENZ inhibited cell proliferation in parental cell lines but showed less effect on proliferation of VCaP‐ENZR and C4‐2B‐ENZR cells as determined with BrdU incorporation (Fig. 1A,B). Both VCaP‐ENZR and C4‐2B‐ENZR cells expressed significantly higher levels of AR‐V7 protein and mRNA compared to the parental cells (Fig. 1C,D).

Clinical studies reveal that a high level of AR‐V7 is an independent predictor of shorter overall survival, shorter PSA progression‐free survival, and shorter clinical progression‐free survival [24]. A comparison of levels of AR‐V7 in clinical samples of CRPC from bone marrow metastases revealed that some CRPC samples had ‘markedly higher’ levels of AR‐V7 compared to levels in androgen‐deprived VCaP cells [25]. VCaP cells carry an amplified AR gene [26] and express an 11‐fold increase in AR mRNA compared to LNCaP cells [27]. In addition, VCaP cells have elevated mRNA expression of AR‐V7 and other AR variants [28] compared to C4‐2B cells. Consistent with these reports, we found enhanced FL‐AR and approximately 10‐fold increase in AR‐V7 expression in VCaP‐ENZR cells compared to C4‐2B ENZR cells (Fig. S1).

Sequencing the FL‐AR from VCaP‐ENZ revealed no unique mutation compared to the sequence for FL‐AR in the parental VCaP cells (data not shown). The F876L mutation reported to confer resistance to ENZ [29, 30, 31] was not detected. RNA‐seq data for C4‐2B ENZR cells deposited at GEO site (#GSE120006) revealed no new mutations compared to parental cells [32].

The role of AR‐V7 in the mechanism of ENZ resistance was investigated by knocking down AR‐V7 by siRNA targeting Exon 3b and measuring effects on cell proliferation with treatment to ENZ. The efficiencies of siRNA knockdown of AR‐V7 are shown by western blot analyses (Fig. 1E,F). Data for proliferation were normalized to clarify how knocking down AR‐V7 affects sensitivity to ENZ. Knockdown of AR‐V7 restored sensitivity of both VCaP‐ENZR and C4‐2B‐ENZR cells to ENZ (Fig. 1E,F). In the absence of ENZ, knockdown of AR‐Vs resulted in approximately a 13% decrease in proliferation of VCaP‐ENZR cells, whereas in C4‐2B‐ENZR, there was a decrease in approximately 50% (see plots for each cell line that were not normalized). These data imply that C4‐2B‐ENZR cells are more dependent on AR‐Vs compared to VCaP‐ENZR cells. Knockdown of AR‐Vs resulted in VCaP‐ENZR cells becoming sensitive to a low dose of ENZ (0.5 µm) (Fig. 1E) which may suggest a shift in reliance to FL‐AR in these cells. C4‐2B‐ENZR cells were less sensitive to ENZ with AR‐V7 knockdown relative to VCaP‐ENZR cells. These results indicate overexpression of AR‐V7 conferred resistance to ENZ.

### 3.2 Effect of EPI‐7170 in combination with ENZ on cell proliferation in ENZ‐resistant prostate cancer cells

**Fig. 2:**
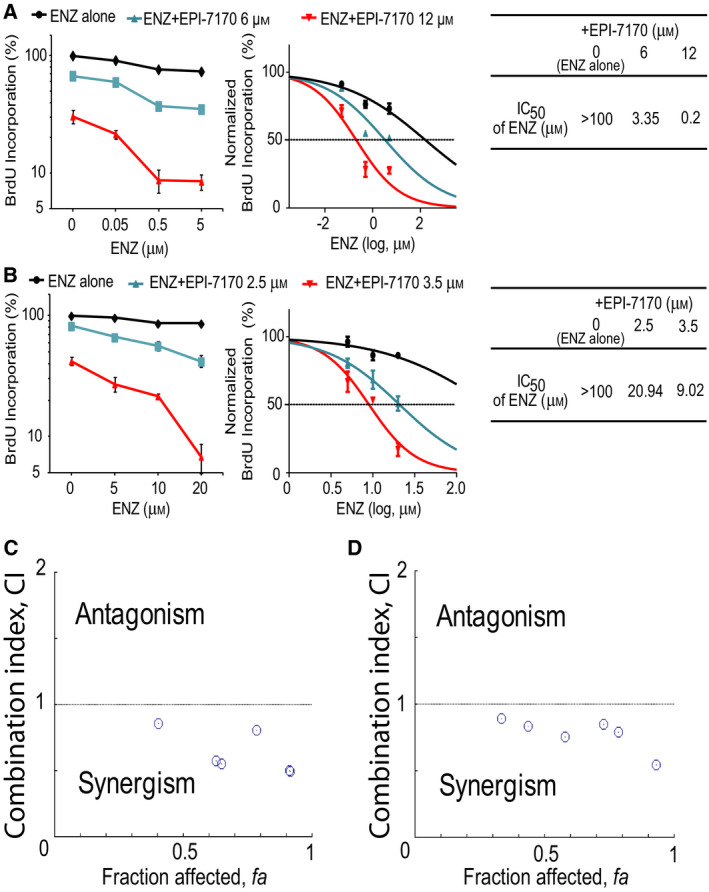
Synergistic interaction between EPI‐7170 and ENZ in ENZR cells. (A) VCaP‐ENZR and (B) C4‐2B‐ENZR cells were treated with DMSO, ENZ, EPI‐7170, or its combination at the indicated concentrations for 5 days (for VCaP‐ENZR) or 2 days (for C4‐2B‐ENZR), and cell proliferation was analyzed by BrdU incorporation assay. IC50s of ENZ alone or in the presence of EPI‐7170 are shown in the tables. (C) Combination index (CI) versus fraction‐affected (Fa) plots for VCaP‐ENZR and (D) C4‐2B‐ENZR were generated by CompuSyn software. CI < 1 represents synergism, CI = 1 additive effect and CI > 1 antagonism. Values represent the mean ± SEM (*n* = 3).

Next, we assessed the effect of ENZ in combination with EPI‐7170 on cell proliferation. EPI‐7170 is a second‐generation analog of ralaniten that has approximately 8‐ to 9‐fold improved potency relative to ralaniten as measured using androgen‐induced PSA‐luciferase reporter and BrdU proliferation assays in C4‐2B‐ENZR cells (Fig. S2). We hypothesized that a combination of an inhibitor of the AR‐NTD with a LBD inhibitor may have improved antitumor activity in ENZ‐resistant cells that express AR‐Vs. VCaP‐ENZR (Fig. 2A) and C4‐2B‐ENZR (Fig. 2B) cells were treated with increasing concentrations of ENZ and EPI‐7170, either alone or in combination. EPI‐7170 alone inhibited cell proliferation in both VCaP‐ENZR and C4‐2B‐ENZR cells as expected. Importantly, EPI‐7170 enhanced the effect of ENZ as evidenced by a lower IC_50_ of ENZ when combined with EPI‐7170. Combination index (CI) values were calculated from the fraction‐affected (Fa; inhibition of cell proliferation) value of each combination using the Chou‐Talalay method to determine potential synergism (CI < 1), additive effect (CI = 1), or antagonism (CI > 1). The CI values were lower than 1 at all the tested concentrations of EPI‐7170 and ENZ (Fig. 2C,D). These data imply a synergistic interaction between EPI‐7170 and ENZ in AR‐V7‐positive cells that are resistant to ENZ.

### 3.3 EPI‐7170 inhibits the transcriptional activity of AR‐V7 in the presence of FL‐AR

**Fig. 3:**
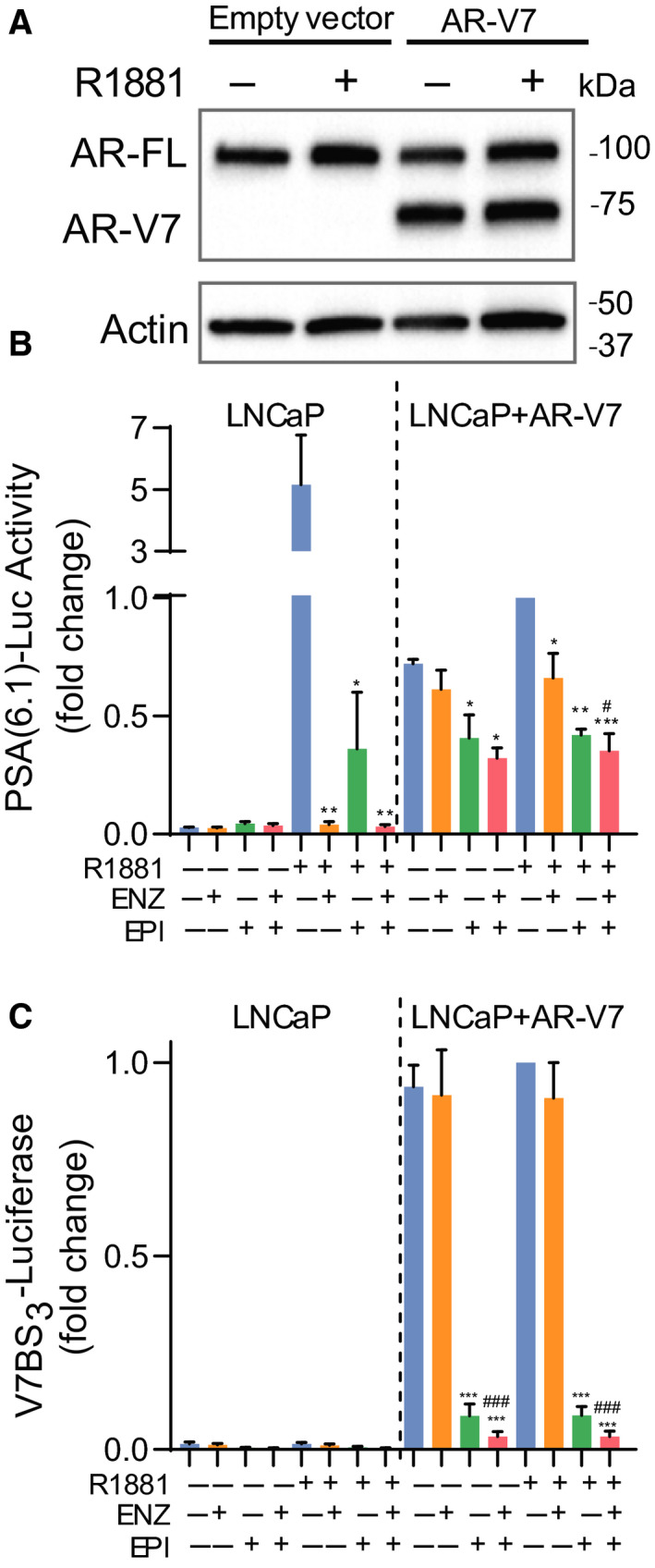
Blocking the transcriptional activities of FL‐AR and AR‐V7. (A) Levels of AR‐V7 ectopically expressed in LNCaP cells were comparable to levels of endogenous AR‐FL. A representative western blot from *n* = 3 independent experiments is shown. (B) PSA(6.1 kb)‐luciferase and (C) V7BS_3_‐luciferase activities in cells treated with DMSO, ENZ (5 µm), EPI‐7170 (5 µm), or the combination in the presence or absence of 1 nm R1881 for 24 h. 1‐fold was set for samples treated with (+) R1881, (−) ENZ, (−) EPI. Statistical significance: * indicates vs DMSO control. # indicates vs ENZ treatment group. n.s., not statistically significant. *P < 0.05; **P < 0.01; ***P < 0.001; ^#^ *P* < 0.05; ^###^ *P* < 0.001. ANOVA test with Tukey's test. Values represent the mean ± SEM (*n* = 3).

To verify that the transcriptional activity of AR‐V7 drives ENZ resistance in the presence of FL‐AR, LNCaP cells were transfected with AR‐V7 expression plasmid or empty vector and then treated with ENZ, EPI‐7170, or combination therapy in the presence or absence of synthetic androgen, R1881. Transcriptional activities of FL‐AR and AR‐V7 were measured using the PSA(6.1 kb)‐luciferase reporter [20], whereas the transcriptional activity of solely AR‐V7 was assessed using the V7BS_3_‐luciferase reporter that is an artificial reporter containing three AR‐V7 binding sites linked in tandem in front of a minimal promoter [33]. Levels of ectopic AR‐V7 relative to endogenous FL‐AR in cell lysates were analyzed by western blot (Fig. 3A). Both ENZ and EPI‐7170 inhibited androgen‐induced PSA‐luciferase activity driven by endogenous FL‐AR. The expression of ectopic AR‐V7 induced both PSA‐ and V7BS_3_‐luciferase activities regardless of androgen. ENZ had no effect on the transcriptional activity of AR‐V7 to induce PSA‐ or V7BS_3_‐luciferase activities, whereas EPI‐7170 inhibited AR‐V7 induced regardless of androgen (Fig. 3B,C). These data confirm that EPI‐7170 blocks both FL‐AR and AR‐V7 transcriptional activities and suggest that a combination therapy of inhibitor to AR‐LBD with an inhibitor to AR‐NTD may be superior to monotherapies in ENZR cells that express both FL‐AR and AR‐Vs.

### 3.4 Effects of EPI‐7170 in combination with ENZ on AR‐FL and AR‐V7 target genes

**Fig. 4:**
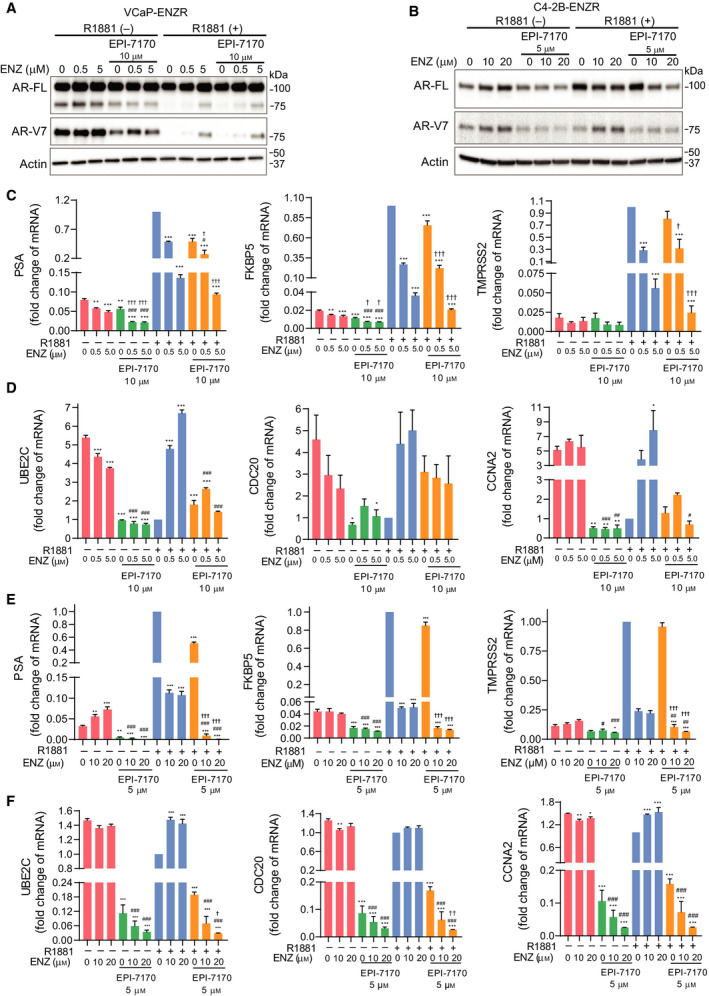
Effect of combined ENZ/EPI‐7170 treatments on expression of FL‐AR and AR‐V7 target genes. (A) Levels of FL‐AR and AR‐V7 proteins in VCaP‐ENZR and (B) C4‐2B‐ENZR cells that were treated with DMSO, ENZ, EPI‐7170, or its combination in the presence or absence of 1 nm R1881 for 2 days. A representative western blot from *n* = 3 independent experiments is shown. (C) mRNA levels of genes regulated by FL‐AR or (D) AR‐V7 in VCaP‐ENZR cells. (E) mRNA levels of genes regulated by FL‐AR or (F) AR‐V7 in C4‐2B‐ENZR cells. Levels of mRNA were normalized to RPL13a with 1‐fold set for samples treated with (+) R1881, (0 µm) ENZ, (0 µm) EPI. Statistical significance: * indicates vs DMSO control. # indicates vs ENZ treatment group. † indicates vs EPI‐7170 treatment group. *P < 0.05; **P < 0.01; ***P < 0.001; ^#^ *P* < 0.05; ^##^ *P* < 0.01; ^###^ *P* < 0.001; ^†^ *P* < 0.05; ^††^ *P* < 0.01; ^†††^ *P* < 0.001. ANOVA test with Tukey's test. Bars represent the mean ± SEM (*n* = 3).

Androgen reduces, while ENZ increases, the expression of AR‐V7 in prostate cancer cells [23]. Consistent with those observations, the levels of AR‐V7 protein were increased with exposure to ENZ in both VCaP‐ENZR and C4‐2B‐ENZR cells (Fig. 4A,B). EPI‐7170 did not increase levels of FL‐AR or AR‐V7 and appeared to antagonize the effects of ENZ in the absence of androgen. Measurement of expression of target genes of FL‐AR (KLK3/PSA, FKBP5, TMPRSS2) and AR‐V7 (UBE2C, CDC20, CCNA2) [34, 35, 36] revealed that ENZ was a more potent inhibitor of androgen‐induced FL‐AR target genes compared to EPI‐7170 but had no effect of V7‐target genes, whereas EPI‐7170 was extremely effective (Fig. 4C–F). Combination of ENZ and EPI‐7170 was significantly more effective than monotherapies for blocking expression of FL‐AR target genes induced by androgen in both ENZR cell lines (Fig. 4C,E). Consistent with the decrease in levels of AR‐V7 protein in response to androgen in VCaP‐ENZR, the expression of AR‐V7 target genes was also suppressed by androgen (Fig. 4D). ENZ treatment increased AR‐V7 target genes, such as UBE2C and CCNA2 in the presence of R1881 (Fig. 4D,F). EPI‐7170 significantly decreased expression of AR‐V7 target genes which were upregulated in the absence of R1881 or with exposure to ENZ (Fig. 4D). In C4‐2B‐ENZR cells, a combination of ENZ with EPI‐7170 was more effective than monotherapies to block expression of AR‐V7 target genes (Fig. 4F). Taken together, levels of AR‐V7 protein and AR‐V7 target genes were upregulated under androgen depletion or ENZ treatment while significantly suppressed with EPI‐7170. These results support that EPI‐7170 can synergize with ENZ to inhibit AR transcriptional activity in ENZR cells that express AR‐V7.

### 3.5 EPI‐7170 inhibited cell growth by cell cycle arrest

**Fig. 5:**
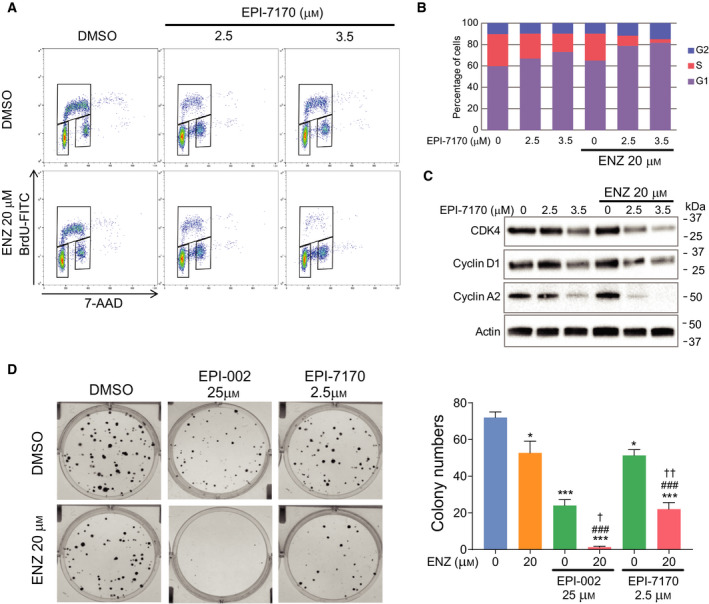
Effects of combination therapy on cell cycle and clonogenic growth. (A) C4‐2B‐ENZR cells were treated with DMSO, ENZ, EPI‐7170, or its combination for 2 days, and cell cycle distribution was analyzed by flow cytometry. Representative bivariate plots of BrdU and 7‐AAD from *n* = 3 independent experiments are shown. (B) The average percentages of cells in each phase of the cell cycle. Data represent the average from *n* = 3 independent experiments. (C) Western blot analysis of cell cycle‐regulated proteins. A representative western blot from *n* = 3 independent experiments is shown. (D) Crystal violet staining of colonies in C4‐2B‐ENZR cells treated with DMSO, ENZ, ralaniten (EPI‐002), EPI‐7170, or the combination for 14 days. Right graph shows the values from three separate experiments. Statistical significance: * indicates vs DMSO control. # indicates vs ENZ treatment group. † indicates vs EPI‐7170 treatment group. *P < 0.05; ***P < 0.001; ^###^ *P* < 0.001; ^†^ *P* < 0.05; ^††^ *P* < 0.01. ANOVA test with Tukey's test. Bars represent the mean ± SEM (*n* = 3).

To better understand the mechanism of synergy between EPI‐7170 and ENZ, their effects on cell cycle in C4‐2B‐ENZR cells were assessed. ENZ (20 µm) monotherapy had little effect on cell cycle, whereas EPI‐7170 (3.5 µm) monotherapy led to an increase in G1 fraction and reduction of S phase. Combination therapy resulted in nearly complete inhibition of DNA synthesis in S phase (Fig. 5A,B). Analysis of some key cell cycle‐related proteins revealed that EPI‐7170 decreased the levels of CDK4, cyclin D1, and cyclin A2 proteins. Combination of EPI‐7170 with ENZ further decreased these levels in spite of ENZ monotherapy having little effect (Fig. 5C). This combination led to levels of cyclin A2 protein that were below our levels of detection. Cyclin A2 is involved in initiation of DNA synthesis, thereby supporting results obtained from cell cycle analysis. Moreover, consistent results were also obtained with the clonogenic assay. Combinations of ENZ (20 µm) with ralaniten (25 µm) or low dose EPI‐7170 (2.5 µm) were more effective than monotherapies in C4‐2B‐ENZR cells (Fig. 5D).

### 3.6 *In vivo* a combination of ENZ and EPI‐7170 has improved antitumor activity

**Fig. 6:**
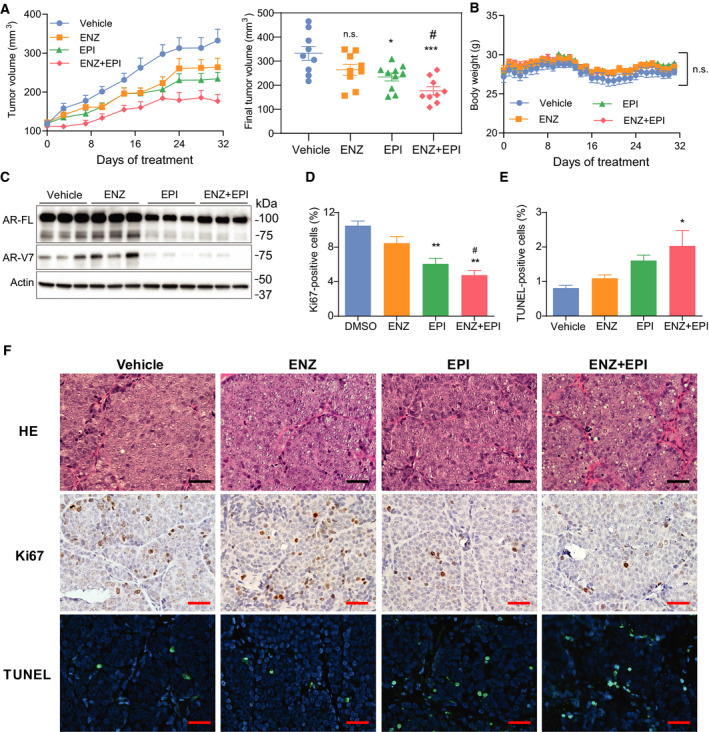
Combination therapy has improved antitumor activity. (A) Tumor volumes of established subcutaneous VCaP‐ENZR xenografts treated with vehicle, ENZ (20 mg·kg^−1^ body weight), EPI‐7170 (30 mg·kg^−1^ body weight), or combination for 31 days (*n* = 9 or 10 for each group). Right graph shows final tumor volume at day 31. Values represent the mean ± SEM. (B) Body weights were measured daily for all animals for the 31 days. Values represent the mean ± SEM. (C) Levels of FL‐AR and AR‐V7 proteins normalized to β‐actin in protein lysates prepared from 3 harvested representative xenografts from each group. (D) Cells that were positive for Ki67 and (E) TUNEL staining were counted from three xenografts per treatment group. Total number of cells counted: 4421 (vehicle, Ki67), 4924 (ENZ, Ki67), 4871 (EPI‐7170, Ki67), 4241 (combination, Ki67), 3459 (vehicle, TUNEL), 3944 (ENZ, TUNEL), 3625 (EPI‐7170, TUNEL), and 3407 (combination, TUNEL). Bars represent the mean ± SEM (*n* = 3). (F) Representative images of staining for hematoxylin and eosin, Ki67 and TUNEL in tumors. Scale bars, 25 µm. Statistical significance: * indicates vs vehicle control. # indicates vs ENZ treatment group. n.s., not statistically significant; *P < 0.05; **P < 0.01; ***P < 0.001; ^#^ *P* < 0.05. *P* = 0.2492 for EPI vs EPI + ENZ. ANOVA test with Tukey's test.

To evaluate the therapeutic efficacy of combining ENZ with EPI‐7170 *in vivo*, VCaP‐ENZR cells were inoculated subcutaneously into male NOD/SCID mice. Mice were castrated and orally dosed daily either with vehicle, ENZ (20 mg·kg^−1^ body weight), EPI‐7170 (30 mg·kg^−1^ body weight), or a combination (ENZ 20 mg·kg^−1^ body weight + EPI‐7170 30 mg·kg^−1^ body weight) for 31 days. EPI‐7170 alone significantly decreased the final tumor volume compared to the controls, whereas ENZ monotherapy did not. The combination of ENZ and EPI‐7170 significantly decreased tumor volume compared to ENZ alone and vehicle treatment (Fig. 6A). However, there was no statistically significant difference measurable by tumor volume between the combination therapy and EPI alone. No significant change in body weight was observed among the treatment groups (Fig. 6B). Consistent with *in vitro* results, protein levels of AR‐V7 were increased in harvested tumors from hosts treated with ENZ (Fig. 6C). EPI‐7170 monotherapy and in combination showed decreased levels of FL‐AR and AR‐V7 in harvested xenografts (Fig. 6C). To examine the mechanism of tumor suppression, we evaluated levels of proliferation and apoptosis. EPI monotherapy and combination therapy inhibited proliferation as indicated by Ki67 staining (Fig. 6D). TUNEL analysis showed induced apoptosis within tumors from mice treated with combination therapy (Fig. 6E). Immunohistochemistry of representative xenografts is shown (Fig. 6F).

## 4 Discussion

AR‐V7 is a promising predictive biomarker for lack of responses to ENZ and abiraterone in CRPC [6, 12, 13, 14, 15]. However, whether AR‐V7 is a key driver of drug resistance or a true therapeutic target is less clear. In this report, we demonstrated the role of AR‐V7 in the development of resistance to ENZ in prostate cancer. Long‐term treatment of ENZ conferred resistance to ENZ accompanied with increased expression of AR‐V7 in VCaP‐ENZR and C4‐2B‐ENZR cells. Importantly, knockdown of AR‐V7 restored sensitivity to ENZ, thereby supporting that AR‐V7 expression contributes to the acquisition of resistance. This was also verified by the inability of ENZ to impact the transcriptional activity of ectopic AR‐V7 in the presence of FL‐AR.

ENZ is a second‐generation AR‐LBD inhibitor with greater potency and no agonist properties [37] that has advanced the treatment of CRPC patients. Furthermore, recent clinical trials have shown significant clinical benefits of early use of second‐generation antiandrogens including ENZ, apalutamide, and darolutamide in patients with nonmetastatic CRPC or metastatic hormone‐sensitive prostate cancer compared to placebo [38, 39, 40, 41, 42]. However, these clinical paradigm shifts with earlier application of second‐generation antiandrogens may result in earlier increased expression of AR‐Vs in prostate cancer patients. Our results show increased AR‐V7 expression after long‐term ENZ treatment leads to resistant to ENZ and are consistent with reported clinical and experimental studies [23, 25, 43]. ENZ acts as a selective pressure leading to drug resistance. Despite the emerging clinical importance of AR‐V7, optimal management of AR‐V7‐positive CRPC still remains to be determined. Chemotherapy might be favored rather than antiandrogens for AR‐V7‐positive CRPC due to the clinical relevance of AR‐V7 status [44, 45]. There is an urgent need to develop new strategies targeting AR‐V7 signaling to overcome drug resistance in CRPC.

We here show ENZ significantly inhibited FL‐AR signaling induced by androgen, while ENZ increased both AR‐V7 protein and expression of AR‐V7 target genes in ENZ‐resistant prostate cancer cells. This indicates that therapies targeting AR‐LBD promote a shift in signaling from FL‐AR to AR‐V7 that contributes to resistance to antiandrogen therapies. Most importantly, upregulated AR‐V7 transcriptional activities were blocked when combined with AR‐NTD antagonist, EPI‐7170. This provides a reasonable explanation for the synergistic antiproliferative effect of combining EPI‐7170 with ENZ on ENZ‐resistant prostate cancer cells that express AR‐V7 as shown in this study. Cell cycle analysis also showed consistent results with ENZ alone having little effect on cell cycle, while EPI‐7170 alone led to G1 cell cycle arrest and combination therapy inhibited DNA synthesis. These data suggest cell cycle arrest was induced by targeting AR‐V7, which is in accordance with previous studies showing that AR‐Vs control cell cycle pathway [46, 47, 48] as well as FL‐AR [49].

Due to the clinical relevance of AR‐V7 in CRPC, several novel agents that indirectly decrease its expression are being investigated and include: quercetin which inhibits heterogeneous nuclear RNA‐binding protein A1 (hnRNPA1) [50]; JQ1, a bromodomain, and extraterminal domain (BET) inhibitor [51]; and niclosamide, an FDA‐approved antihelminthic drug [22]. Consistent with these agents decreasing levels of AR‐V7, they restored sensitivity to ENZ [22, 50, 51]. Here, we showed inhibition of AR‐V7 expression or blocking its transcriptional activity selectively with the AR‐NTD inhibitor EPI‐7170 both restored sensitivity to ENZ. However, synergy was not measurable in vivo at the doses and method of drug delivery used here. The ability to obtain synergy often depends on the concentration ratio of the combined drugs. *In vitro* these concentrations are easily controlled, but in vivo there may vast differences in distribution and pharmacokinetics of the different drugs used in the combination, thereby impeding the optimal concentration ratio within the tumor that are required to obtain synergy [52]. To date, the pharmacokinetics of EPI‐7170 have not been reported but the first‐generation mixture of ralaniten and its analogs (EPI‐001) had a plasma elimination half‐life of 3.27 h in mice at an oral dose of 100 mg·kg^−1^ body weight [20], whereas enzalutamide at an oral dose of 10 mg·kg^−1^ body weight had a half‐life of 15.8 h [53]. Thus, further optimization of dosing based upon pharmacokinetic data may be required to achieve synergy *in vivo*.

Clinical support for the AR‐NTD as a drug target can be drawn from the first in human clinical trial with ralaniten (NCT02606123) in heavily pretreated CRPC patients that had failed ENZ and/or abiraterone. This clinical trial revealed some signs of efficacy with ralaniten as indicated by reduction in serum prostate‐specific antigen (PSA) and stable disease in some patients in spite of having 50× lower Cmin blood levels than necessary for in vitro efficacy [17]. This clinical trial also provided validation of the ralaniten scaffold for developing drugs to treat CRPC. A second‐generation ralaniten analog will be tested in clinical trials in 2020 (NCT04421222).

## 5 Conclusions

In conclusion, this study revealed ENZ treatment leads to increased AR‐V7 expression that confers resistance to ENZ. By directly targeting AR‐NTD to block both FL‐AR and AR‐V7, EPI‐7170 showed synergistic effects in combination with ENZ. This study advances the current understanding of the role of AR‐V7 in the mechanism of resistance to antiandrogens and provides a new translatable therapeutic option targeting AR‐V7 to overcome CRPC.

## Conflict of interest

The authors declare the following competing interests: YH, MDS, KJ, and RJA are inventors of technology which was licensed by the BC Cancer to ESSA Pharma. MDS and RJA have equity and are Scientific Advisors for ESSA Pharma. Their interests were reviewed and are managed by the BC Cancer Agency and University of British Columbia in accordance with its research conflict of interest policies. TT has no competing interests.

## Author contributions

YH and MDS conceived the study, designed the experiments, interpreted the data, and wrote the manuscript. YH performed the biological experiments and analyzed the results. TT sequenced the FL‐AR in VCAP‐ENZR cells. KJ and RJA synthesized EPI‐7170. MDS supervised the study. All authors read and approved the final manuscript.

## Supporting information


**Fig. S1.** Relative protein levels of FL‐AR and AR‐V7 in cell lines. (A) Whole cell protein lysates from cells were run on a SDS‐PAGE gel and then analyzed for levels of FL‐AR (left) and AR‐V7 (right) using antibodies to the AR‐NTD (detects FL‐AR and AR‐Vs) and AR‐V7. Actin was used as a loading control. (B) Quantification of bands shown in “A” for FL‐AR and AR‐V7 normalized with levels of β‐actin that was used as a loading control. The numbers over the bars are the normalized values for AR and AR‐V7 in the two ENZR cell lines. n = 1.

**Fig. S2.** EPI‐7170 has improved potency compared to ralaniten (EPI‐002) to block androgen‐induced AR transcriptional activity and proliferation in C4‐2B‐ENZR cells. (A) Dose‐dependent inhibition of androgen‐induced transcriptional activity of FL‐AR in C4‐2B‐ENZR cells by EPI‐002 or EPI‐7170. C4‐2B‐ENZR cells transfected with the PSA‐luciferase reporter were treated with EPI‐002 or EPI‐7170 for 1hr prior to the addition of R1881 (1nM) for 24 hours. Values represent the mean ± SEM (n = 3). (B) Effects of EPI‐002 or EPI‐7170 on androgen‐induced proliferation of C4‐2B‐ENZR cells. IC50s of EPI‐002 or EPI‐7170 are shown in the table (right). Values represent the mean ± SEM (n = 3).

## Data Availability

The raw data are available from the corresponding author upon reasonable request.
